# Anti-Extraction

**Published:** 1892-01

**Authors:** Wm. N. Morrison

**Affiliations:** St. Louis, Mo.


ARTICLE IV.

### ANTI-EXTRACTION.

BY WM. N. MORRISON, D. D S., ST. LOUIS, MO.

Read before the St. Louis Dental Society, 1883.

Yearly there are extracted, by the bushel, teeth and roots firmly set in the jaws, susceptible of repair and capable of performing a duty and service ten times greater than that of the best artificial substitutes. A patient with the toothache cannot take a calm, sensible view of the situation, of the irreparable injury, not only through the loss of the member, but by change of position the others will take, and the unavoidable loss of the use of its opposing fellow. Out of one hundred teeth extracted daily in this city, ninety-nine should not be extracted, but should be carefully and painlessly cleansed from soft decay at the margins (not over the pulp) and filled with some non-conducting cement and kept filled, imperfectly though it may be.

There are many persons, most ladies, who wear jewels and pearls and expensive clothing to beautify their exterior, while they trust their pearls of inestimable value to the hands of the most inexperienced cheap dentist, and get his cheapest wares at his cheapest prices, and then go abroad and boast of the amount economized in their dental bills, when one glance at their faces and one zephyr from their mouths is most convincing that their dental services were most dearly bought at the expense of their beauty and health. The worthy cheap dentist has a fruitful field and can do an immense amount of good if he would confine himself to legitimate cheap dentistry, without mentioning an arbitrary fee for gold work, not equal, in many cases, to half of what the material used would cost.

The patient, young or old, should be told how to brush and pick and rinse his teeth, and this fact impressed upon him; that teeth decay only from the outside and for want of proper care and cleanliness. Dentists make their living out of their patient's neglect and ignorance of the correct laws of hygiene, just as physicians do.

With our advanced knowledge of the worth of good natural teeth, and the present improved instruments and facilities for treating sensitive cavities, in all positions of the teeth, in a comparatively painless manner, and the numerous materials for filling such cavities, each good in its place, there is no truth in the saying that any tooth or root cannot be filled. All turnkeys, forceps and extracting screws, as constructed to remove firm teeth or roots, are barbarous relics of the Inquisition. For years I have extracted teeth or roots only when they were so loose that they could be removed with the thumb and finger, and I most heartily wish every other member of the profession would adopt that rule. They would be gratified at the conservation and restoration nature can accomplish. There should be no artificial dentures made by the future dentist. The artificial leg and arm maker could increase his business with as much justice by recommending the amputation of all rheumatic and neuralgic limbs by the aid of gas, and start out his victims with his substitutes which bear the same relation to the natural limb that the best dental substitutes bear to the natural teeth and shorten their lives by many years.

Consider for a moment thirty-two natural teeth, in normal jaws, capable of being brought together with a pressure of four hundred pounds to the square inch upon a morsel of meat (tough Texas);then the involuntary swallowing of the juice, leaving the solid fibres dry and empty to absorb the secretion like a sponge from the glands whose ducts open at that part of the mouth. The morsel so saturated with normal saliva is almost digested without the action of the stomach upon it. Such persons rarely realize that they have a stomach. Then contrast with that the poor old receptacle of dyspepsia's remedies. The sallow complexion, hollow eyes and hollow jaws, with artificial teeth half the size of the natural ones, and only twenty-eight in number, resting on a spongy gum instead of being anchored three-quarters of an inch in the bone, covering the membranes and tissues with which the food ought to come in contact, brought together with feeble force in an up and down motion only. Mastication, in the true sense, cannot be performed with artificial teeth. Little squares of meat are rolled around in the mouth, and may have a few holes punched into them, but are sent nearly whole into the stomach, thus compelling that organ to perform two offices—that of mastication as well as that of digestion. This is a subject the public have entirely overlooked; and the mass of dentists, if they have given it any thought and know its direful results, do not exert their best energies to correct the evil. Legislative exactness will not correct the evil so long as the physicians can extract teeth *ad libitum* and dental shambles advertise that all their employes are dental graduates. In all State bills for the regulations of the practice of dentistry there is an exempting clause which states, "Nothing in the bill shall prevent physicians from extracting teeth." The average physician knows no more about treating a case of common toothache than a last year's bird's nest. If he has talent for surgery, he will extract; if he is of the timid *materia medica* type, he will write a prescription and send his victim to the nearest drug store, never looking into the cavity of the teeth to see what produces the ache, which comes always from pressure on the pulp (or nerve), impacted food or gas from decomposed pulp tissue confined in the pulp canal. If from the first cause, relief may be given by simply removing the food, cleansing the cavity and closing it with a cotton saturated with sandaric varnish. If the ache is caused by the mephitic gas, on opening into the pulp chamber will give immediate relief. All the decomposed pulp tissue should be removed and the canal stopped temporarily with cotton and creosote. If it is a case of abscess, open at the same time as a simple abscess on any other part of the body.

The same general rule applies to the children's or deciduous teeth. If they are carefully cleansed and kept from decay, they will drop in their proper time, as do the leaves in antumn. When the pulps are dead and the crowns occlude against their opposing fellows, I snap the crowns off with excising forceps, and keep the roots ground down, but leave them in the alveolus as wedges, which increase the arch. More cases of irregularity of the permanent teeth can be traced to extraction of the deciduous teeth than to any other cause.

Now, as long as the law on this subject stands as it is, the very point that should be protected is thrown open, and every one is allowed to extract teeth, regardless of any knowledge on the subject. Make it punishable by fine for any one to extract a firm tooth or root, and welcome every one or every instrument and material that will save teeth, and you will, by these means promote health and prolong life to this city and to the State of Missouri.

Dr. W. N. Conrad: Dr. Morrision's paper to-night is the best I have ever heard him read,* barring a few things, such as "never extract teeth until you can remove them with the thumb and finger." Common sense must be used in all such things. All know that teeth *must* be extracted in an ordinary practice. Sometimes teeth must be extracted as soon as erupted. If we absolutely refuse to extract teeth, we are not doing our whole duty, and we must sometimes yield to our patients when we would prefer not.—*Ohio State Journal.*

---

* Dr. Morrison is very proud of the paper, and now agrees with the estimation put upon it by Dr. Conrad, but at that time thought he was getting a little taffy from Dr. C.—ED. *Archivis.*

